# Comparison of Characteristics of Breast Cancer Detected through Different Imaging Modalities in a Large Cohort of Hong Kong Chinese Women: Implication of Imaging Choice on Upcoming Local Screening Program

**DOI:** 10.1155/2022/3882936

**Published:** 2022-10-31

**Authors:** Yik Shuen Chan, Wai Ka Hung, Lok Wa Yuen, Ho Yan Yolanda Chan, Chiu Wing Winnie Chu, Polly Suk Yee Cheung

**Affiliations:** ^1^Department of Imaging & Interventional Radiology, Prince of Wales Hospital, 30-32 Ngan Shing Street, Shatin, Hong Kong SAR, China; ^2^Department of Imaging & Interventional Radiology, The Chinese University of Hong Kong, Shatin, Hong Kong SAR, China; ^3^Hong Kong Breast Cancer Foundation, 22/F, Jupiter Tower, 9 Jupiter Street, North Point, Hong Kong SAR, China; ^4^Breast Health Clinic, CUHK Medical Centre, 9 Chak Cheung Street, Shatin, Hong Kong SAR, China

## Abstract

**Background:**

We compared the clinico-radio-pathological characteristics of breast cancer detected through mammogram (MMG) and ultrasound (USG) and discuss the implication of the choice of imaging as the future direction of our recently launched local screening program.

**Methods:**

Retrospective study of 14613 Hong Kong Chinese female patients with histologically confirmed breast cancer registered in the Hong Kong Breast Cancer Registry between January 2006 and February 2020. Patients were classified into four groups based on the mode of breast cancer detection (detectable by both mammogram and ultrasound (MMG+/USG+), mammogram only (MMG+/USG−), ultrasound only (MMG−/USG+), or not detectable by either (MMG−/USG−). Characteristics of breast cancer detected were compared, including patient demographics, breast density on MMG, mode of presentation, tumour size, histological type, and staging. Types of mammographic abnormalities were also evaluated for MMG+ subgroups.

**Results:**

85% of the cancers were detectable by MMG, while USG detected an additional 9%. MMG+/USG+ cancers were larger, more advanced in stage, often of symptomatic presentation, and commonly manifested as mammographic mass. MMG+/USG− cancers were more likely of asymptomatic presentation, manifested as microcalcifications, and of earlier stage and to be ductal carcinoma in situ. MMG−/USG+ cancers were more likely seen in young patients and those with denser breasts and more likely of symptomatic presentation. MMG−/USG− cancers were often smaller and found in denser breasts.

**Conclusion:**

Mammogram has a good detection rate of cancers in our local population. It has superiority in detecting early cancers by detecting microcalcifications. Our current study agrees that ultrasound is one of the key adjunct tools of breast cancer detection.

## 1. Introduction

According to the World Health Organization (WHO), breast cancer is the world's most prevalent cancer with 7.8 million women alive with breast cancer diagnosed within the past 5 years—with 2.3 million being diagnosed in 2020 and 685,000 lives lost in the same year [1]. Breast cancer is also cancer with the highest incidence and mortality rate in the Asia-Pacific region, with 839,000 new cases in 2018 and over 286,000 breast cancer deaths [2]. In China, Japan, and Korea, breast cancer ranked first in age standardised (world) incidence rates in female amongst all cancers, at 39.1, 76.3, and 64.2 per 100,000, respectively, in the year 2020 [3]. Breast cancer is also the most common cancer and third leading cause of cancer-related deaths of female population in Hong Kong according to the most updated data published by Hong Kong Cancer Registry [4]. The WHO Global Breast Cancer Initiative aims to reduce global breast cancer mortality by 2.5% annually in order to prevent 2.5 million lives lost to breast cancer from 2020 to 2040, with one of the key factors being timely diagnosis [1].

In 2014, the WHO published a position paper on screening for breast cancer with mammography (MMG) [5]. Mammography is the only modality shown to reduce cancer deaths by early detection, with early landmark studies based on a Caucasian population and emerging evidence in the Asian population as well [6, 7]. It is, however, also known that breast cancer detection is limited by the density of the breast, which is higher in the Asian population [8–10]. A systematic review has found that adjunctive ultrasound (USG) detects additional cancer by 0.3–7.7 cancer/1000 examinations (median 4.2) [11]. Emerging evidence has revealed that primary screening USG has comparable sensitivity, specificity, and cancer detection rate to primary screening MMG for women with dense breasts [12]. There is constant debate regarding the use of MMG and USG either alone or in conjunction, for breast cancer detection in a screening setting.

Worldwide, many countries such as the United States, United Kingdom, Australia, Finland, and the Netherlands have adopted population-based breast screening by mammography, with variations in age of women included and screening interval. Mammography is similarly considered the modality of choice for breast screening in Asian countries like South Korea and Japan. In China, the screening program is age-based, stratifying patients to the use of ultrasound and/or mammography.

In Hong Kong, the government recently launched a two-year pilot risk-based screening program since September 2021, targeting women aged between 44 and 69 with a certain combination of risk factors. In this pilot program, eligible women (namely, women found to have higher than average risk) attending designated government clinics are referred for biennial mammography screening, paying a subsidised fee. Ultrasound may be additionally arranged if necessary. Prior to this, the screening practice in Hong Kong had been self-initiative, opportunistic screening, with a heterogeneous selection of imaging modalities. At this very initial stage of introduction of a breast screening program, before a population-based universal screening is confirmed, it is vital to review what is the best imaging modality tailored to our local population to guide our next step of treatment.

To the best of our knowledge, there is limited published data regarding the head-to-head comparison of clinico-radio-pathological characteristics of MMG-detected and USG-detected breast cancers, especially in Asian population. In the current study, we leveraged the data from a large existing database consisting of histological confirmed female breast cancer patients registered with the Hong Kong Breast Cancer Registry over a period of 14 years. We sought to compare the clinico-radio-pathological characteristics of MMG-detected and USG-detected breast cancers and explore the implication of these findings on the future direction of local breast screening programs.

## 2. Materials and Methods

This is a retrospective descriptive study. All the data were retrieved from the Hong Kong Breast Cancer Registry.

### 2.1. The Database

The Hong Kong Breast Cancer Registry was established to collect comprehensive local data on breast cancer from patients' self-report and their medical records. The data were collected at 62 public and private hospitals and clinics widely distributed throughout Hong Kong during the study period. During this period, no universal screening program was in place. The database included patients with different methods of cancer detection. Of the 14613 patients included in the final analysis, the majority of the patients discovered their cancers through self-detection by chance (80.9%, *n* = 11823) followed by a much smaller number of patients with cancer revealed upon mammogram screening (8.9%, *n* = 1330), screening by breast self-examination or clinical breast examination (2.7%, *n* = 389), and through other imaging modalities such as ultrasound and MRI (0.8%, *n* = 116) or incidental surgery (0.8%, *n* = 116), while a small portion of patients (3.9%, *n* = 574) did not have their mode of detection recorded. Majority of patients underwent mammogram and/or ultrasound, but overall, the imaging modality was utilised, and therefore, the data included in the registry were heterogeneous, including but not limited to mammography, ultrasound, and MRI.

### 2.2. The Imaging

All mammography and ultrasound were performed in public and private radiology facilities throughout Hong Kong. All radiology facilities were staffed by radiologists and radiographers qualified in mammography training under the criteria and standard set by the Hong Kong College of Radiology. The mammographic reporting adopted a single-read approach.

### 2.3. Study Population

**Figure 1:**
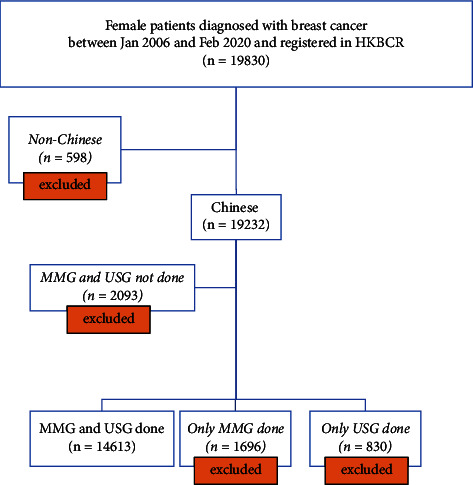
Flowchart of patient selection.

A total of 19830 female patients were diagnosed with breast cancer from January 2006 to February 2020 and registered with the Hong Kong Breast Cancer Registry (HKBCR). The exclusion criteria included patients who were non-Chinese (*n* = 598) and patients who did not have complete set of MMG and USG examination as baseline imaging (*n* = 4619). 14613 Chinese female patients formed the final cohort (Figure 1).

### 2.4. Data Extraction

Data retrieved from Hong Kong Breast Cancer Registry included patients' age at diagnosis, mode of presentation (asymptomatic or symptomatic), clinical tumour size, histological type, and cancer stage at presentation according to the latest TNM version by American Joint Committee on Cancer at the year of diagnosis. Radiological data retrieved included breast density on mammography and types of mammographic abnormalities for MMG+ subgroups.

Results of mammogram and ultrasound were graded by the latest versions of the Breast Imaging-Reporting and Data System (BI-RADS) at the year of diagnosis, with scores of 4-5 considered positive for diagnosis of breast cancer in our analysis. Based on the BI-RADS scheme, breast densities were divided into four categories—(a) almost entirely fat, (b) scattered fibroglandular tissue, (c) heterogeneously dense, and (d) extremely dense, with increasing proportion of fibroglandular tissue from category a to category d. Dense breast density was defined as category c and d.

### 2.5. Study Group Definition

Patients were stratified into four groups based on whether breast cancer was detected from MMG and/or USG results. The (MMG+/USG+) group included cases detectable by both mammogram and ultrasound, the (MMG+/USG−) group included cases which were detectable by mammogram only, the (USG+/MMG−) group included cases detectable by ultrasound only, and (MMG−/USG−) group included cases not detectable by either mammogram or ultrasound.

### 2.6. Statistical Analysis

The chi-square test was applied to compare categorical clinico-patho-radiological variables, including breast density on MMG, mode of presentation, histological type, and stage of breast cancer amongst the four study groups and the MMG findings between the two groups with positive mammographic findings. Continuous variables such as the age of patients and clinical tumour size amongst the four groups were analyzed using the Welch ANOVA test. If part of the data was missing for a particular patient, for example, cancer stage, patients were excluded from the analysis for that particular category but still included in the rest of the analysis. The number of cases included in each analysis is stated in the respective tables. *P* value of <0.05 is considered statistically significant.

Statistical analyses were performed using IBM SPSS Statistics 19 (SPSS Inc., Chicago, IL, United States).

## 3. Results

A total 14,613 patients were included in the study. Amongst those with age documented (*n* = 14443), the majority were presenting at the age of 50–59 (*n* = 4756, 32.5%), followed closely by those presenting at age of 40–49 (*n* = 4602, 31.5%) (Table 1).

In the study population, symptomatic presentation was much more common than asymptomatic presentation (81.1% vs. 15.0%) (Table 1). Majority of patients had dense breasts, i.e., category c and d (*n* = 7852, 77.4%), with the prevailing breast density type being heterogeneous density (*n* = 7099, 70.0%) (Table 2).

### 3.1. Characteristics of the MMG+/USG+ Group

In this group, majority of patients had positive findings on both mammography and ultrasound, irrespective of mode of presentation. The tumours in this group had the largest mean tumour size of 2.51 cm when compared with the other groups (*p* < 0.001) (Table 3). The most common MMG+ abnormality was mammographic mass alone constituting 46.9% (*p* < 0.001), followed by mammographic mass with microcalcifications at 27.1% (*p* < 0.001) (Table 4). For histopathology, most of these tumours were invasive, with a majority being invasive ductal carcinoma (79.9%, *p* < 0.001). For TNM staging, the cancers were more advanced in stage compared with other groups, with high proportion of stage II cancers (41.6%, *p* < 0.001).

### 3.2. Characteristics of the MMG+/USG− Group

In this group, majority of patients had asymptomatic presentation (61.0%, *p* < 0.001). For MMG+ abnormality, a higher proportion of these tumours presented with microcalcifications only (71.4%, *p* < 0.001). For histopathology, DCIS was the most common pathology (50.0%, *p* < 0.001), and accordingly, stage 0 was the most common cancer grade (52.4%, *p* < 0.001) in TMN staging.

### 3.3. Characteristics of the MMG−/USG+ Group

In this group, majority of patients had symptomatic presentation (78.2%, *p* < 0.001). The patients were generally of younger age (54.8% with age <50) and had the highest percentage of dense breast density on MMG (85.2%, *p* < 0.001) as compared with other subgroups. Ultrasound detected small tumours (1-2 cm) that were occult on MMG.

### 3.4. Characteristics of the MMG−/USG− Group

In this group, majority of patients had dense breast density on MMG (82.9%, *p* < 0.001). The tumours were the smallest in size (mean 1.71 cm, *p* < 0.001), with majority of tumours being ≤2 cm when compared with other groups.

## 4. Discussion

This study attempts to evaluate which imaging modality would best serve our predominant Chinese population in our local breast cancer screening program by analysing the clinico-radio-pathological differences of breast cancers detected through different imaging modalities with reference to territory-wide data collected from the Hong Kong Breast Cancer Registry in last 14 years. Through the analysis of cancers detected via different imaging modality combinations, we wish to shed light on what cancers we may be able to capture with different screening imaging modality combinations. Such head-to-head comparison of breast cancer detected with different imaging modalities was rarely addressed in current literature.

The majority of the tumours in our study (85.3%) were detectable by mammogram, implying mammography plays a major role in breast cancer detection for our local population regardless of mammographically denser breasts. This, of course, agrees with all the forefathers in the study of mammography as a screening tool through high quality systemic review and meta-analyses [13]. The tumours detectable only on mammography (i.e., MMG+/USG−) gave us insight about the characteristics of the group of patients that will benefit from a mammography rather than ultrasound screening program. The most common mammographic feature in this group was microcalcifications. DCIS was the most common pathology, and, unsurprisingly, most patients were at stage 0 and often asymptomatic. Indeed, mammogram is sensitive to microcalcifications, irrespective of breast density, which may be the only presenting imaging feature of early stage cancer, especially DCIS. The use of mammography is thus invaluable in population-wide breast screening, and if mammography is not a part of the screening program, cases of DCIS may be missed. In our locality, mammography is widely available, generally acceptable and affordable. Mammography screening is also well-supported by literature, making the choice to adopt a screening program with mammography a straightforward decision.

On the other hand, we found that a greater proportion of the tumours were detectable by ultrasound (92%) than by mammography (85.3%). An additional 9% of the tumours, which were mammographically occult, could be detected by ultrasound, and a majority (75.8%) of these were confirmed to be invasive cancers. This is in keeping with many studies confirming additional cancer detection by ultrasound in mammographically negative patients [12, 14–16]. These mammographic occult tumours detected by ultrasound are seen across all age groups, with the highest significance for those between 40 and 49—age group which constituted almost 32% of our study population. This is intriguing as the above observation prompts the question of whether ultrasound would be more effective in our population for breast cancer detection than mammography. A multicenter randomised controlled trial in China concluded ultrasound to be superior to mammogram in detecting breast cancers in high-risk Chinese women [17]. Another advantage of ultrasound is its acceptability by patients. Despite the fact that mammography is already generally accepted by the public, ultrasound remains superior in acceptability as there is less discomfort. Asian countries are known to have a low participation rate in screening programs, and fear of pain is one of the factors constituting barriers to screening [18]. Shen et al. found that patients in the ultrasound-only arm of their breast screening trial had a higher follow-up rate than those in the mammography-only or combined (mammography and ultrasound) group [17]. At the same time, adherence to a screening program is a key to achieving a sustainable result with mortality improvement. A study by Park et al. found that overall mortality risk was lower for patients with annual compared with biennial mammography [19]. Further investigation is required to see if similar results would apply to ultrasound-based screening. The major drawbacks of ultrasound as a screening modality are its higher recall rate and higher false positive rate [16, 20]. How this translates into cost-effectiveness is beyond the scope of our discussion. At present, the primary use of ultrasound remains as an adjunctive tool.

The shift in the paradigm of breast imaging has added to the complexity of choosing the optimal imaging regimen. The emergence of 3D mammography, i.e., digital breast tomosynthesis, in recent years, poses a possible solution to the issue of screening dense breasts with mammogram, with a superior cancer detection rate and lower recall rate and a comparable radiation dose nowadays to digital mammography alone [21]. At present, no significant difference has been identified in the additional cancer detection rate with adjunctive ultrasound for 2D mammography and 3D mammography [22]. The maturing AI software may also increase our ability to capture cancer. A diagnostic study for AI in a retrospective simulated screening setting found the highest cancer detection rate by teaming up radiologists and AI [23]. A recently published retrospective study by Kim et al. also found AI to be able to detect mammographically occult cancers in dense breasts [24]. With the rapid development of AI software, its application or integration into the breast screening program is anticipated. Breast MRI and the emerging contrast-enhanced mammography are currently limited to the assessment of high-risk women at best. The increasing research in optimisation of MRI sequence, including uses of ultrafast sequences, DWI, and abbreviated MRI protocols, as well as increased availability of these modalities, also paves a new road for future screening options.

Furthermore, our study solidifies the need for a population-wide screening program in Hong Kong. Breast cancer in Hong Kong is not just a disease of the older age group. We found the prevailing majority of breast cancer patients in our locality were between the ages of 40 and 59. The majority of the patients in this study presented symptomatically, in keeping with a lack of a territory-wide screening program. Most tumours in our study cohort were detectable on both mammogram and ultrasound at the time of presentation. The tumours also tended to be larger in size and of later stage, commonly being invasive ductal carcinoma. Activation of a screening program is an important step in identifying early cancer during an asymptomatic state, thus allowing early intervention and the subsequent improvement of cancer-related morbidity and mortality. Currently, the local breast cancer screening program is a two-year pilot program with a risk-based approach, and we root for at least sustaining, if not expanding the screening program in the future.

Our study leveraged the power of a large existing database of breast cancer patients in Hong Kong, which is extremely useful in providing an insight into the breast cancers in the Asian/Chinese population. It is, however, inevitably limited by its retrospective nature, as part of the data required for analysis was incompletely collected. This resulted in a variation in the total number of patients involved in different feature analyses. Nonetheless, the large number of patients available for analysis still served as an invaluable local data to reflect the characteristics of breast cancer in Hong Kong. Caution has to be taken when applying our results to a screening population as our study took place before the launch of a breast screening program in Hong Kong, and the majority of the patients were symptomatically detected. It is expected that the screen-detected tumours will have a different clinicopathological profile from our current study group. And as discussed previously, the potential benefit of digital breast tomosynthesis (DBT), which is currently widely available worldwide, including in Hong Kong, was not investigated in our study as this imaging technique had limited accessibility in our locality during the time when data were collected. In a real-life situation, cost-effectiveness of the screening regimen also has to be taken into consideration.

Nevertheless, our study shed light on the direction and potential appropriate imaging methods of breast cancer screening program for our local population. More input from professional groups is required to answer the question in full, including but not limited to the selection of the most optimal screening protocol for our local population, focusing on the potential of mortality reduction by ultrasound, and consideration of adjunct ultrasound or even alternative to ultrasound screening for a selected group of women for maximizing cost-effectiveness.

## 5. Conclusion

Mammogram plays a major role in breast cancer detection in Chinese women and is able to detect the majority of breast cancer at presentation. The mammogram also shows superior ability in detecting microcalcifications despite high breast density in Chinese women, rendering it particularly useful for DCIS and stage 0 cancer detection. In our cohort, ultrasound was found to have a slight superior detection rate of breast cancer than mammogram, in particular for detection of invasive types of cancers prevailing in patients who were young or with dense breasts. Mammogram and ultrasound are shown to have complementary roles in achieving early breast cancer detection in our large local cohort, and their combined use should be taken into consideration for future breast screening programs.

## Figures and Tables

**Table 1:** Characteristics of study population.

	*N* (total = 14613)	%
Age		
<40	1233	8.4
40–49	4602	31.5
50–59	4756	32.5
60–69	2758	18.9
≥70	1094	7.5
Unknown	170	1.2

Cancer stage		
Stage 0	1752	12.0
Stage I	4606	31.5
Stage II	5455	37.3
Stage III	2042	14.0
Stage IV	294	2.0
Not classified	464	3.2

Mode of presentation		
Symptomatic	11848	81.1
Asymptomatic	2191	15.0
Unknown	574	3.9

Mode of cancer detection		
MMG+/USG+	12131	83.0
MMG+/USG−	335	2.3
MMG−/USG+	1311	9.0
MMG−/USG−	836	5.7

**Table 2:** Age and breast density distribution of study population by group.

	MMG+/USG+		MMG+/USG−		MMG−/USG+		MMG−/USG−		*P* value
	*N*	%	*N*	%	*N*	%	*N*	%	
Age group (*n* = 14443)									<0.001
<40	949	7.9	25	7.6	149	11.5	110	13.4	
40–49	3571	29.8	133	40.3	560	43.3	338	41.1	
50–59	4029	33.6	119	36.1	380	29.4	228	27.7	
60–69	2458	20.5	45	13.6	153	11.8	102	12.4	
≥70	990	8.2	8	2.4	52	4.0	44	5.4	

Breast density (*n* = 10147)									<0.001
Nondense	2011	(23.9)	50	(20.1)	135	(14.8)	99	(17.1)	
Fatty	1216	14.5	35	14.1	69	7.6	58	10.0	
Scattered density	795	9.5	15	6.0	66	7.2	41	7.1	
Dense	6397	(76.1)	199	(79.9)	777	(85.2)	479	(82.9)	
Heterogeneous density	5813	69.1	173	69.5	682	74.8	431	74.6	
Extreme density	584	6.9	26	10.4	95	10.4	48	8.3	

**Table 3:** Clinical and pathological tumour characteristics of study population by group.

	MMG+/USG+		MMG+/USG−		MMG−/USG+		MMG−/USG−		*P* value
	*N*	%	*N*	%	*N*	%	*N*	%	
Mode of presentation (*n* = 14039)									<0.001
Symptomatic	10081	86.6	127	39.0	990	78.2	650	80.3	
Asymptomatic	1557	13.4	199	61.0	276	21.8	159	19.7	

Clinical tumour size (*n* = 10544)									<0.001
**Mean**	**2.51**	**1.89**	**1.77**	**1.71**					
≤1.00 cm	542	5.9	29	24.6	146	16.9	105	22.9	
1.01–2.00 cm	3906	42.9	51	43.2	503	58.1	246	53.6	
2.01–5.00 cm	4175	45.9	35	29.7	204	23.6	100	21.8	
>5.00 cm	479	5.3	3	2.5	12	1.4	8	1.7	

Histological type (*n* = 14198)									<0.001
IDC	9406	79.9	126	38.2	900	70.5	413	50.7	
ILC	378	3.2	6	1.8	57	4.5	21	2.5	
Mixed IDC and ILC	49	0.4	0	0.0	10	0.8	0	0.0	
DCIS	1008	8.6	165	50.0	188	14.7	250	30.7	
Others	936	7.9	33	10.0	121	9.5	131	16.1	

Cancer stage (*n* = 14149)									<0.001
Stage 0	1087	9.3	175	52.4	215	16.6	275	33.8	
Stage I	3569	30.5	106	31.7	589	45.5	342	42.0	
Stage II	4868	41.6	43	12.9	380	29.4	164	20.2	
Stage III	1899	16.2	10	3.0	101	7.8	32	3.9	
Stage IV	284	2.4	0	0.0	9	0.7	1	0.1	

IDC, invasive ductal carcinoma; ILC, invasive lobular carcinoma; DCIS, ductal carcinoma in situ.

**Table 4:** MMG features observed in patients with positive MMG.

	MMG+/USG+		MMG+/USG−		*P* value
	*N*	%	*N*	%	
MMG features (*n* = 9844)					
Mass only	4482	46.9	45	15.3	<0.001
Microcalcifications only	1908	20.0	210	71.4	<0.001
Mass and microcalcifications	2590	27.1	26	8.9	<0.001
Architectural distortion only	208	2.2	6	2.0	0.874
Asymmetric density only	362	3.8	7	2.4	0.210

## Data Availability

The data used to support the findings of this study are retrieved from Hong Kong Breast Cancer Registry and are available from the corresponding author upon request.

## Abbreviations

MMG: Mammogram
USG: Ultrasound
HKBCR: Hong Kong Breast Cancer Registry
WHO: World Health Organization
DCIS: Ductal carcinoma in situ
DBT: Digital breast tomosynthesis.
